# Association between Pesticide Profiles Used on Agricultural Fields near Maternal Residences during Pregnancy and IQ at Age 7 Years

**DOI:** 10.3390/ijerph14050506

**Published:** 2017-05-09

**Authors:** Eric Coker, Robert Gunier, Asa Bradman, Kim Harley, Katherine Kogut, John Molitor, Brenda Eskenazi

**Affiliations:** 1 School of Public Health, University of California, Berkeley, CA 94703, USA; gunier@berkeley.edu (R.G.); abradman@berkeley.edu (A.B.); kharley@berkeley.edu (K.H.); kkogut8@berkeley.edu (K.K.); eskenazi@berkeley.edu (B.E.); 2 College of Public Health and Human Sciences, Oregon State University, Corvallis, OR 97331, USA; john.molitor@oregonstate.edu

**Keywords:** neurodevelopment, pesticides, organophosphates, mixtures, carbamates, pyrethroids

## Abstract

We previously showed that potential prenatal exposure to agricultural pesticides was associated with adverse neurodevelopmental outcomes in children, yet the effects of joint exposure to multiple pesticides is poorly understood. In this paper, we investigate associations between the joint distribution of agricultural use patterns of multiple pesticides (denoted as “pesticide profiles”) applied near maternal residences during pregnancy and Full-Scale Intelligence Quotient (FSIQ) at 7 years of age. Among a cohort of children residing in California’s Salinas Valley, we used Pesticide Use Report (PUR) data to characterize potential exposure from use within 1 km of maternal residences during pregnancy for 15 potentially neurotoxic pesticides from five different chemical classes. We used Bayesian profile regression (BPR) to examine associations between clustered pesticide profiles and deficits in childhood FSIQ. BPR identified eight distinct clusters of prenatal pesticide profiles. Two of the pesticide profile clusters exhibited some of the highest cumulative pesticide use levels and were associated with deficits in adjusted FSIQ of −6.9 (95% credible interval: −11.3, −2.2) and −6.4 (95% credible interval: −13.1, 0.49), respectively, when compared with the pesticide profile cluster that showed the lowest level of pesticides use. Although maternal residence during pregnancy near high agricultural use of multiple neurotoxic pesticides was associated with FSIQ deficit, the magnitude of the associations showed potential for sub-additive effects. Epidemiologic analysis of pesticides and their potential health effects can benefit from a multi-pollutant approach to analysis.

## 1. Introduction

Several epidemiologic studies have shown associations between biomarkers of prenatal exposure to pesticides and poorer childhood neurodevelopment [1,2,3,4,5,6,7,8,9,10]. We have also shown in the Center for the Health Assessment of Mothers and Children of Salinas (CHAMACOS) birth cohort study that the average of two measurements of organophosphate pesticides (OPs) dialkyl phosphate (DAPs) metabolites in maternal urine collected during pregnancy was associated with decrements in cognitive development of 7-year-old children living in an agricultural community [11]. However, although this biomarker potentially represents exposure to a multitude of OPs, it does not represent all OPs used and it does not indicate exposure to specific pesticides. To address this limitation, we previously investigated prenatal residential proximity to agricultural pesticide-use using California’s unique database of Pesticide Use Data (PUR), which provides the poundage (kg), date, and location (to one-square-mile sections) of specific pesticides (active ingredient) applied; we found significant adverse associations between cognitive development in children at 7 years of age and specific agricultural pesticides or grouped pesticide classes, namely oxydemeton-methyl, acephate, pyrethroids, neonicotinoids, and Mn-fungicides [12]. However, due to the inherent limitations with conventional multiple regression techniques—such as limited capacity to handle large numbers of highly correlated exposures—our previous analyses and others’ [1,9,13] have relied on multiple tests of association with neurodevelopment for individual pesticides or pesticide groups, and we therefore have yet to evaluate the potential neurodevelopmental effects of joint exposure to several different individual pesticides with different mechanisms of toxicity. 

Since agricultural populations are potentially exposed through a variety of routes to a combination of pesticides that exhibit varying toxicities and modes of action [14], a modeling approach is needed which limits multiple tests of association (that enhance type 1 error rate) while also handling multicollinearity. Since many pesticides are neurotoxic, albeit of varying potency (even within chemical pesticide classes) [15], and some have similar mechanisms of action [15,16,17,18], there is also a potential for either additive or non-additive effects given different combinations of pesticide exposures. Statistical approaches have recently emerged to facilitate analysis of the combined health effects of joint exposure to multiple environmental chemical exposures [19,20,21,22,23,24,25,26,27].

Agricultural communities present an opportunity to investigate the potential health effects of exposure to multiple pesticides. California presents a strong case study, due both to its relatively high volume of pesticide use—approximately 85 million kg in 2014 [28]—and the fact that 100% of all agricultural pesticide use is reported to the state’s Department of Pesticide Regulation [29]. Past environmental and biomonitoring studies that have utilized PUR data have shown positive correlations between nearby reported agricultural pesticide use and pesticide concentrations measured in outdoor air and house dust [30,31,32,33]. We have also found that the CHAMACOS mothers—during pregnancy—had higher OP urinary metabolites levels compared to the general U.S. population [34] and that living near agricultural fields is related to higher levels of urinary OP metabolites at younger ages in the children [35]. To date, however, little is known about the spatial patterning of joint use near residents in agricultural communities and whether different joint use patterns may be more strongly associated with health outcomes. 

In this study, we investigated the joint distribution of potentially neurotoxic pesticide-use profiles during pregnancy with measures of childhood cognition. We included in this analysis pesticides not previously considered because they do not devolve to DAPs and/or have no known biomarker. We also explored whether there was a distinct spatial patterning of some of the combinations of pesticide-use that showed stronger associations with child cognition. To accomplish this, we employed a novel statistical method known as Bayesian profile regression (BPR) [23] to analyze pesticide use profiles that are estimated from prenatal residential proximity to reported agricultural pesticide use. The BPR approach is based on well-established Bayesian Dirichlet process mixture modeling techniques [36,37,38,39] and is capable of: (1) accounting for the collinearity of the exposure data inherent with agricultural pesticide use; (2) appropriately handling model uncertainty in cluster assignment and number of clusters used; and (3) drawing inference on health associations by linking profiles of exposure with a health outcome of interest. In this way, we employ a joint exposures approach to identify and characterize the important clusters of prenatal pesticide profiles that are asociated with deficits in childhood cognition. We also aimed to identify individual pesticides that are most strongly associated with childhood cognition when considering joint exposure by applying Bayesian Kernel Machine Regression (BKMR), a data-adaptive method that allows for fitting multiple correlated exposures jointly into the same model, and evaluating each parameter’s relative importance across the model space.

## 2. Materials and Methods

### 2.1. Study Population: CHAMACOS Cohort

The CHAMACOS study is a longitudinal birth cohort study examining pesticide and other environmental exposures in the agricultural Salinas Valley, California. Detailed information about the study is presented in Eskenazi et al. [40]. Participant mothers were recruited and enrolled from October 1999 to October 2000 from clinics serving the low-income residents. Women were eligible if they were ≥18 years of age, ≤20 weeks of gestation, were eligible for MediCal, spoke English or Spanish, and were expecting to deliver at the county hospital. Of 601 mothers enrolled, 537 were followed to delivery. Twins (*n* = 10) and children with medical conditions that could affect their neurodevelopment assessment (*n* = 4) were excluded from the analysis (one child each with Down syndrome, autism, deafness, and hydrocephalus). Up to as many 330 children were followed through the 7-year of age for neurodevelopmental assessment. Children were included in our analysis only if we knew their maternal residence during pregnancy for a minimum of 75 days for each of two or more trimesters (*n* = 283), if they completed each component of the neurodevelopmental assessment at age 7 years (*n* = 257), and if they had prenatal measurements of DAPs (*n* = 255 with DAPs), since DAPs were an important covariate in our statistical analysis [12]. This resulted in a final study population of 255 (see Supplementary Materials, Table S1 for the study population characteristics). Written informed consent was obtained from all women and verbal assent from all children at age 7 years; all research was approved by the University of California, Berkeley, Committee for the Protection of Human Subjects prior to commencement of the study (Ethical Approval Code: 2010-03-949). 

### 2.2. Characterization of Prenatal Pesticide Use near Maternal Residences

We characterized potential exposure based on prenatal residential proximity (≤1 km radius) to agricultural pesticide applications. Pesticide use information was obtained from the state PUR database (described above). These methods have been previously described [12,41]. Briefly, three measurements using a global positioning system at the maternal residence were collected to determine latitude and longitude [32]. We created a 1-km buffer radius around the location of each maternal residence and weighted the amount of pesticide active ingredient applied within each 2.59 square-km (or one square-mile) section by the proportion of land area within the 1-km buffer. We selected a 1-km buffer around residences because our previous work has shown this distance to better explain the variation in observed house dust concentrations for most of the agricultural pesticides sampled in homes (compared to smaller distance-based buffers) [30]. To calculate pesticide use over an entire pregnancy, we first determined reported pesticide use during each trimester period based on at least 75 days of known residential location for each trimester as the criterion for calculating an entire pregnancy average. For mothers with at least two trimesters that met this criteria, we summed and then averaged across the entire pregnancy by dividing by the number of trimesters included. We selected 30 neurotoxic insecticides and two manganese (Mn)-based fungicides a priori-based on evidence from human or animal studies [42,43,44,45,46] that had agricultural use during our study period, including fifteen OPs, six carbamates, eight pyrethroids, and one neonicotinoid. Of these pesticides, up to 15 pesticides from five different chemical classes, including seven OPs (oxydemeton-methyl, acephate, malathion, diazinon, dimethoate, chlorpyrifos, and naled), two carbamates (methomyl and thiodicarb), four pyrethroids (permethrin, cyhalomethrin, cypermethrin, and esfenvalerate), one Mn-based fungicide (maneb), and one neonicotinoid (imidacloprid) met our criteria for sufficient use within 1 km of prenatal residences to be included in the final analysis. We set our criteria of sufficient use for each pesticide if the median estimate across our study population was above zero and where there was at least a two-fold difference between the lower 25th percentile and 75th percentile of the distribution for a pesticide-use estimate (to ensure there is sufficient contrast in exposure). 

### 2.3. Assessment of Cognitive Development

Our study outcome was cognitive ability of the 7-year-old children determined by Full Scale Intelligence Quotient (FSIQ) assessed using the Wechsler Intelligence Scale for Children, 4th edition (WISC-IV) [47]. We selected FSIQ as our outcome since our previous studies show consistent associations between FSIQ and prenatal urinary DAPs [11] and prenatal residential proximity to reported agricultural pesticide use [12,41]. A single bilingual psychometrician conducted all assessments. These were administered in the dominant language of the child as determined by an oral vocabulary subtest [48], with 67% completing the WISC-IV testing in Spanish and 33% in English. The WISC-IV comprises four separate domains, including verbal comprehension, perceptual reasoning, working memory, and processing speed, which were combined to derive a FSIQ score [47]. WISC-IV scores were then standardized against U.S. population-based norms for English- and Spanish-speaking children.

### 2.4. Covariates

We selected household-level and individual-level covariates a priori that have been controlled for in our previous publications that examined pesticides and IQ at age 7 years [11,12]. Household-level covariates included the Home Observation for Measurement of the Environment—short form (HOME) score (continuous) [49] and household poverty level (dichotomous < poverty level vs. ≥ poverty level) assessed at the child’s seven-year visit. Child covariates included age at assessment (continuous), language of assessment, and sex. Maternal covariates included educational attainment level (≤6th grade vs. >6th grade), maternal intellectual abilities based on Peabody Picture Vocabulary Test completed at 6 months postpartum (continuous) [50], country of origin (Mexico vs. other), at risk for depression at the child’s seven-year visit (≥16 on CES-D), and average prenatal urinary total DAPs concentrations (log 10 scale). Prenatal DAPs were measured in urine samples collected at two time points during pregnancy (median = week 13 at first time point and median = week 26 for second time point) [11]. We control for prenatal DAPs because we observed an association with seven-year IQ in a previous analysis [11] and because DAPs remained significantly associated with IQ even when nearby OP pesticide use based on PUR data was included in the model [12]. In addition, controlling for total DAPs was considered important because we wanted to control for other potential sources (i.e., diet and take-home from resident farmworkers) of prenatal OP pesticide exposures that cannot be ascertained from PUR data, and was justified because DAPs were not correlated with PUR exposure estimates.

### 2.5. Statistical Analysis

#### 2.5.1. Bayesian Profile Regression

We utilized Bayesian profile regression (BPR) because applications of different pesticides are highly correlated, and BPR is useful for analyzing such data structures in relation to an outcome. Compared to many other clustering or dimension reduction procedures (i.e., k-means or principle component analysis), BPR offers important advantages. First, BPR addresses uncertainty in cluster assignment by means of Bayesian modeling in conjunction with Markov chain Monte Carlo (MCMC) estimation [51]. Second, BPR, is based on well-established Dirichlet Process based clustering methods [36,52] and assumes an infinite number of possible clusters, so the analyst need not specify beforehand the number of clusters to be used, whereas other clustering procedures (e.g., k-means) typically assume a fixed number of clusters and that individual observations may be in only a single cluster as the algorithm proceeds. Third, BPR flexibly links the exposure profile clusters with a health outcome via a disease sub-model that partially supervises the clustering assignment in a unified manner. Fourth, while BPR accounts for uncertainty in clustering, it also employs a dissimilarity matrix to derive an interpretable “best” clustering related to the joint distribution of exposures. The full BPR procedure addresses uncertainty related to this “best” partition by model averaging through all partitions (clusterings) produced by the stochastic estimation process. Large, stable clusters will generally be associated with consistent and overall high cluster assignment probabilities and associated with narrower posterior credible intervals related to cluster parameters, while smaller, less stable clusters will generally be associated with wider interval estimates.

Briefly, for each individual, i, we define a pesticide use profile as, $\;x_{i}=\left(x_{i1},{\;x}_{i2},\;\ldots ,{\;x}_{\mathrm{iP}}\right)$, which includes the entire suite of prenatal pesticide use estimates, with pesticide covariates denoted as $x_{p}$, p =1,…,P for individual i, with *P* = 15 indicating the total number of potential exposures. Each exposure was categorized into discrete categorical “exposure” covariates, where covariate $x_{ip}$ has $K_{p}$ number of categories. Here, K = 4 for each pesticide covariate, since we used discrete pesticide-use quartiles for each pesticide included in BPR. These pesticide-use estimates were categorized due to the high skewness in their distributions for each pesticide analyzed. 

In the full BPR analysis, the categorical covariate mixture model is fit in a unified manner with a response vector, $Y_{i}$, such that the contribution of the covariate data to a response variable depends on the cluster assignment. Thus, the relationship between joint pesticide exposure and FSIQ is cluster dependent. We modeled the response, FSIQ, as a continuous variable with normally distributed errors. Additionally, a set of global fixed effect confounders, $W_{i}$, were fit as control variables on the response. Continuous confounders were centered on their means to better facilitate MCMC convergence and to enable us to appropriately interpret cluster effect estimate posterior distributions provided in the full BPR model output. All BPR analyses were implemented in R (R Core Team, Vienna, Austria) using the *PReMiuM* package with default priors, and MCMC output was checked for convergence using trace plots of betas for the fixed effects ($W_{i}$) [53]. For more details on BPR, see [23,53,54,55].

For each cluster of pesticide-use patterns, henceforth denoted as “pesticide profiles”, we derive the posterior distributions from the MCMC iterations of the fully adjusted expected FSIQ score (for baseline values of discrete and centered continuous confounders). We focus our Bayesian inference on the difference in adjusted FSIQ score for each c^th^ cluster compared with the adjusted FSIQ scores for the cluster that showed the lowest pesticide-use (defined as the pesticide profile cluster group that resulted in posterior distributions with the highest proportion of observations in the lowest quartile of exposure across all of the pesticides). Comparison with a low exposure ‘baseline’ group is a common method of risk characterization in environmental epidemiology studies where no participants in the cohort study are ‘unexposed’ [56].We report on the posterior mean and quantile FSIQ of each cluster and the posterior probabilities for a deficit in FSIQ compared to the low exposure cluster. 

BPR was also rerun by excluding the FSIQ outcome, as we have done in our previous work published using BPR [21], as a sensitivity analysis to assess whether the exposure profile clustering patterns is different compared with including the FSIQ outcome in the model. We applied the same exact model parameters between runs (20,000 burn in and 200,000 MCMC sweeps). 

To better understand the role of particular pesticides driving the clustering, we used the variable selection option in the *PReMiuM* package. The variable selection options, which are comprised of either a binary [37] or continuous [57] selection weighting methods, allows the model to exclude an exposure from influencing the clustering procedure if an exposure exhibits a very low probability of being involved in the clustering patterns, further emphasizing a data-driven (non-parametric) approach to clustering. Specifically, we implemented variable selection with the “Continuous” option which utilizes a latent variable taking on values in (0,1) which informs the contribution of the variable in question in supporting a mixture distribution [53,57]. Using a Bayesian framework for variable selection has been shown to be particularly helpful within the context of a large number of correlated covariates because it appropriately handles model uncertainty [58,59]. Here we evaluate the posterior inclusion probabilities for each pesticide to derive their cluster support.

#### 2.5.2. Characterizing Pesticide-Use Profile Clusters

To help characterize the pesticide use profile clusters, we used the output from the full BPR analysis of the “best” clustering assignments to assume a type of “hard” cluster assignment for each study participant (thus ignoring uncertainty in cluster assignment). We evaluated the joint distributions of empirical values for pesticide-use estimates and further summarized the predominant pesticides within each cluster for these “best” clusters. Next, we summarized the empirical distributions of child FSIQ scores corresponding to these “hard” clusters that were derived from BPR. Finally, using the *PReMiuM* package, we fit a multivariate linear regression model to evaluate the fully adjusted association between pesticide profile clusters and FSIQ compared to the lowest pesticide profile cluster as a “baseline exposure” reference group, properly accounting for cluster uncertainty in both assignment and number of clusters. The pesticide profile clusters that were derived from the sensitivity analysis, which excluded the outcome in the BPR analysis, were also fit using multivariate linear regression as a second stage to the analysis so that inference may be compared between the fully Bayesian risk characterization when the outcome is included in the BPR analysis.

#### 2.5.3. Pesticide Importance by Bayesian Kernel Machine Regression

We then applied a novel statistical modeling and estimation method called Bayesian Kernel Machine Regression (BKMR) [19] to distinguish the relative importance of single pesticides across all possible models when fitting the study pesticides jointly. The primary benefits of BKMR when fitting a joint exposure model comes from its uniform analysis of an exposure-response surface by way of exposure profiles, as well as the adaptive data-driven nature of the kernel estimation procedure that allows for model non-additivity and exposure-response non-linearity, along with variable selection to help identify key components of a mixture potentially responsible for the outcome. In our implementation, we chose the hierarchical variable selection because several of our exposure measures are highly correlated. In an effort to deal with collinearity, the hierarchical variable selection option does not allow exposures that are grouped together to enter into the same model [19]. BKMR variable selection is akin to Bayesian variable selection and thus computes posterior inclusion probabilities (PIPs) to compare the relative ranking of each variable being selected into the model. For BKMR with hierarchical variable selection, we specified the groups based on the chemical classes, and alternatively based on the correlation structure of the exposure variables in a sensitivity analysis. We also used the same fixed effects as in profile regression [19]. While the output from BKMR is rich with information for inference, we focus our presentation of the results from BKMR on PIPs to illustrate the relative ranking of variable importance for each pesticide class as well as each pesticide within a particular class of pesticides. This analysis thus acts as a supplement to profile regression to help illustrate the variables that may be most important with respect to FSIQ.

### 2.6. Mapping Pesticide Use Profiles

We mapped the point locations (ArcGIS version 10.2, ESRI, Redlands, CA, USA) of participant maternal residences to evaluate the spatial patterning of the assigned clusters [21] as well as the cumulative pesticide use patterns. Specifically, a kernel density plot was applied to the spatial data of the pesticide profiles with stronger associations with FSIQ deficits in order to represent the residential point locations for a particular profile as a smoothed surface to characterize areas where point locations are either more or less concentrated. Finally, we applied both global and local Moran’s I tests to determine whether cumulative pesticide-use patterns or child FSIQ values were spatially clustered. 

## 3. Results

### 3.1. Pesticide Use Summaries

**Figure 1:**
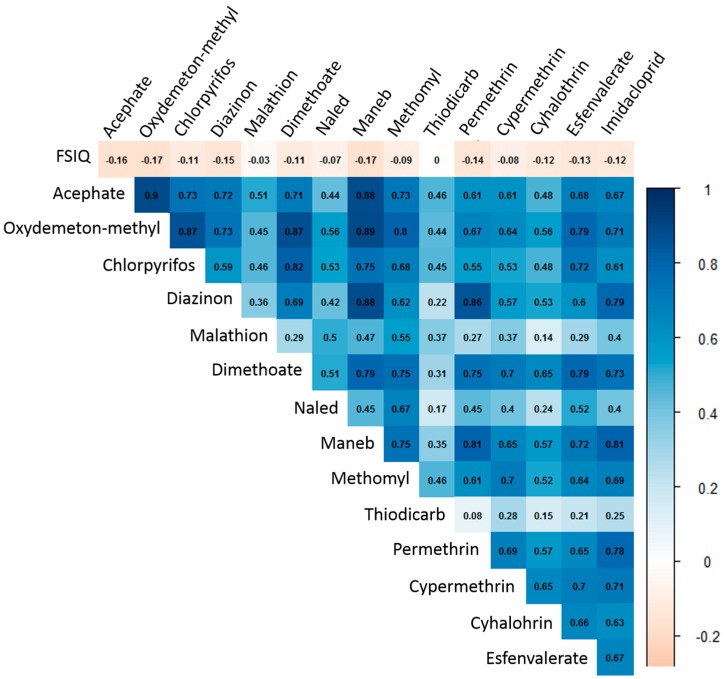
Spearman’s correlations between individual estimates of prenatal pesticide use and correlations with Full-Scale Intelligence Quotient (FSIQ).

The median of the cumulative (summed) estimates for the targeted neurotoxic pesticides within 1 km of maternal residence was 164 kg (interquartile range (IQR) = 68, 356) (Table 1). Several of the pesticide-use proximity estimates showed high to very high between-pesticide Spearman’s correlations (Figure 1). The OPs showed particularly high Spearman’s correlation with each other (ρ range: 0.71, 0.90), except for malathion and naled, which showed only moderate correlations with the other OPs (ρ < 0.56). Several of the OPs were also moderately to highly correlated with other potentially neurotoxic pesticides from other chemical classes, including maneb, methomyl, imidacloprid, and permethrin (see Figure 1). 

### 3.2. Pesticide Use Profile Clusters

Each pesticide in the profile regression resulted in high clustering inclusion probabilities (range: 0.987, 0.998). Profile regression partitioned the joint distribution of individual pesticide estimates into eight pesticide profile clusters. Of these eight pesticide profile clusters, cluster profile 1 (CP1) resulted in the largest median cumulative pesticide-use (median_cluster1_ = 671 kg; IQR = 472, 1030), followed by cluster profiles CP2 > CP7 > CP4 > CP5 > CP8 > CP3 > CP6. 

**Figure 2:**
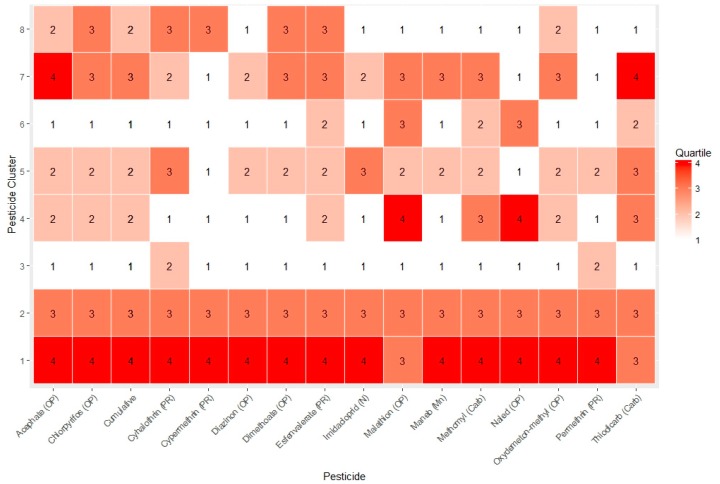
Heat map displaying which quartile the median pesticide use estimate falls into for each “best” cluster from the BPR (Bayesian profile regression) analysis. Each column represents a pesticide while each row represents a cluster profile (CP1 through CP8). The cumulative column refers to the summation of all pesticides estimates for each observation and therefore represents the cumulative pesticide level for each cluster.

**Figure 3:**
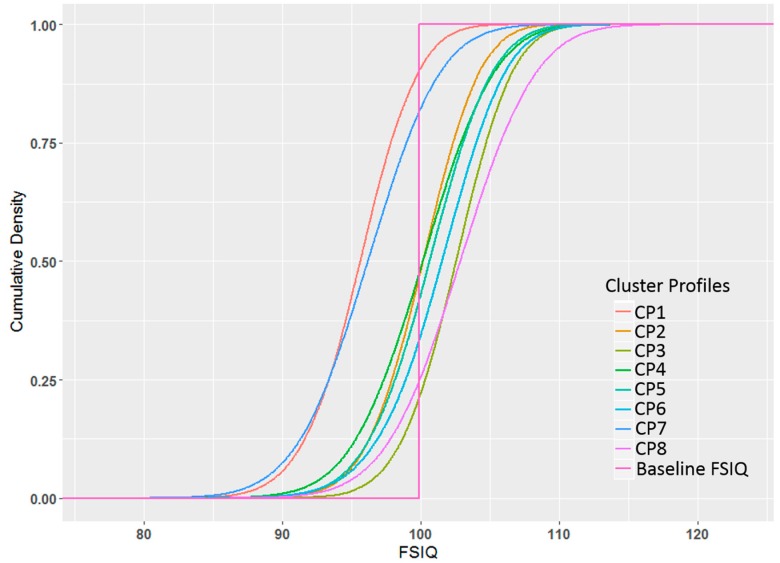
Cumulative probability density plots of cluster-specific posterior adjusted FSIQ distribution (compared to baseline FSIQ). Baseline FSIQ was determined by computing an unweighted overall average FSIQ across all clusters at each iteration (mean = 99.8), however, when calculating a weighted overall average FSIQ (weighted by cluster sizes), the difference between Baseline FISQ and weighted Baseline FSIQ is marginal (<0.5).

Since each “best” pesticide profile cluster consists of joint distributions for all pesticides studied, we used a heuristic approach to characterize predominant pesticides within each pesticide profile cluster (see Figure 2 in a manner similar to Liverani et al. [60]). This heuristic was based on calculating the median for observations within a cluster that fall into a particular quartile. For each pesticide profile cluster, we characterized a specific pesticide as either (1) “very high” potential exposure if the within cluster pesticide-use median of observations is in the fourth quartile (darkest red tiles in Figure 2); (2) “moderately high” if the within cluster pesticide-use median of observations is in the third quartile for a given pesticide (lighter red tiles in Figure 2); (3) “moderately low” if the within cluster median of observations is in the second quartile (lightest red tiles in Figure 2); and (4) “very low” if the within cluster pesticide-use median of observations are in the first quartile (white tiles in Figure 2). For example, the highest cumulative pesticide profile (CP1) was characterized as very high (red tiles in Figure 2) for 13 of 15 pesticides studied, including oxydemeton-methyl, acephate, diazinon, dimethoate, naled, maneb, methomyl, permethrin, cypermethrin, imidacloprid, chlorpyrifos, and cyhalothrin, and esfenvalerate (dark red boxplots in Figure 2), while CP1 pesticide-use was moderately high for thiodicarb and malathion only (light red tiles in Figure 2). CP3 showed the highest frequency for pesticide-use estimates in the lowest quartile (white tiles, 13 of 15 pesticides). As shown in Figure 2, the BPR partitioned the joint pesticide distributions effectively because each of the clusters resulted in joint pesticide levels across the 15 pesticides that are clearly quite distinct from one another. The fully Bayesian posteriors of the joint distributions for pesticide-use profiles, which still retain the cluster uncertainty propagated by the MCMC algorithm, can be viewed in the Supplementary Materials (Figure S1) which similarly suggest effective partitioning. The posterior distributions from the MCMC iterations indicate that CP1 is elevated for all pesticides while CP3 is low for all pesticides, which is largely in line with the clustering summary presented in Figure 2.

### 3.3. FSIQ Results

Table 2 shows the empirical FSIQ summary statistics overall and stratified by the eight “best” pesticide profile clusters (CP1–CP8) derived from the full BPR. The overall mean FSIQ was 103.8 (95% confidence interval (CI): 102.1, 105.6). CP1 (mean = 98.6, 95% CI: 95.1, 102.2) and CP7 (mean = 101.4, 95% CI: 94.4, 108.4) had the lowest unadjusted mean FSIQs, while all other clusters had unadjusted FSIQ scores that were either similar to or higher than the overall mean FSIQ (Table 2). CP3 resulted in posterior distributions with the highest proportion of observations in the lowest quartile of pesticide-use estimates across all of the pesticides and was therefore selected as the reference group to help facilitate risk characterization of these pesticide profile clusters. 

The posterior distributions of the adjusted expected FSIQ estimates from profile regression, when holding confounders at baseline (zero), are shown in the form of cumulative probability density plots in Figure 3 and are further summarized in Table 3. Pesticide profile clusters CP1 and CP7 are characterized by adjusted FSIQ posterior distributions mostly below the unweighted overall average expected FSIQ (shifted to the left), while all other clusters are characterized by adjusted FSIQ posterior distributions that are either centered around the average or above the average FSIQ (Figure 3). For CP1 and CP7, the joint posterior probabilities for a FSIQ deficit compared with the lowest pesticide profile’s (CP3) FSIQ was 99% and 97%, respectively (Table 3). The overall difference in adjusted FSIQ for CP1 and CP7 compared with CP3’s adjusted FSIQ were −6.9 (95% credible interval: −11.7, −2.1) and −6.4 (95% credible interval: −13.1, 0.5), respectively. Other clusters that showed high posterior probabilities for adjusted FSIQ below that of CP3 included CP2 (83%), CP5 (77%), and CP4 (76%), while all other pesticide profiles resulted in posterior probabilities <50%.

### 3.4. Comparison of BPR Clustering When Excluding the Outcome

As a sensitivity analysis, we also ran the BPR procedure to assess potential differences in “hard” clustering patterns when the outcome is excluded from the model (hence no outcome feedback) versus the analysis presented in Table 3 above, where the outcome was included in the full BPR. While there is extensive overlap in study participants between the clustering when comparing BPR with and without the outcome, it is clear from Table 4 that clustering patterns differed when the outcome was excluded. For example, CP1 for both runs remained the highest pesticide profile cluster, however, four of the participants in CP1 from the analysis that included the outcome were partitioned into a different cluster from the analysis that did not include the outcome. A similar pattern was seen for CP7 from the analysis that included the outcome, with four participants being partitioned into a different cluster from CP7 for the analysis that did not include the outcome (although CP7 was still a relatively high pesticide profile cluster in both analyses). Meanwhile, the lowest exposure pesticide profile cluster from both analyses included the same participants. Table S2 in the Supplementary Materials shows regression coefficients that are similar to the results that included the outcome in the fully Bayesian analysis. Specifically, CP1 and CP7, which are mostly comprised of the same study participants (with the exception of the difference already noted), resulted in similar deficits of FSIQ relative to the lowest pesticide profile cluster. However, associations were not as strong as compared with the outcomes in the fully Bayesian output (likely due to larger sample size in these fully Bayesian clusters). As noted by the authors of the *PReMiuM* package, the MCMC algorithm generally has an easier time splitting rather than merging clusters. Our results suggest that including the outcome may have played an additional role in the MCMC algorithm to help merge observations into a cluster that would have otherwise been split into different clusters. In addition, the unified fully BPR analysis that included the outcome did not require a second step to the regression analysis and also avoided some of the issues related to multiple tests of association that the second-stage linear regression analysis does require when the outcome is excluded from BPR. 

### 3.5. Spatial Patterns of Risk and Potential Exposure

**Figure 4:**
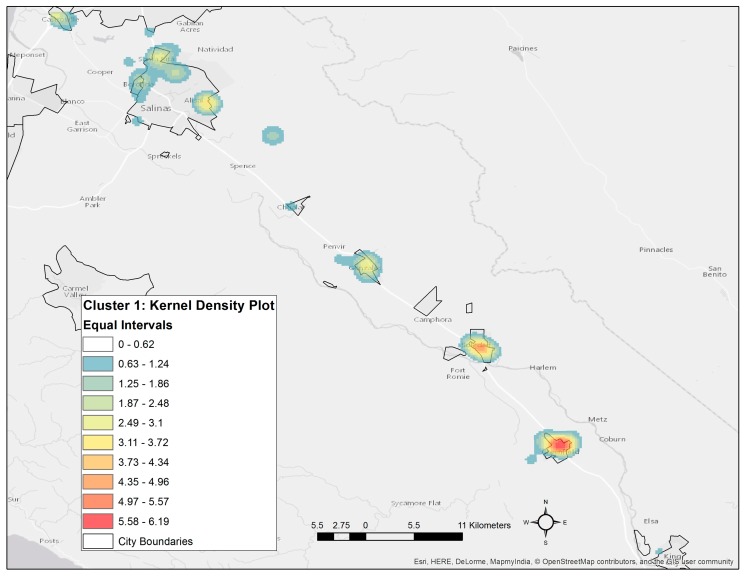
Map of Salinas Valley displaying a kernel density surface indicating the point density of CP1 residences. Higher numbers (orange to red) represents higher spatial point density.

A kernel density plot shown in Figure 4 indicates that maternal residential locations for CP1, the highest risk pesticide profile, are concentrated in the southern areas of the Salinas Valley and along the outskirts of the City of Salinas. The geostatistical pattern for CP7 could not be fully evaluated due to the relatively small number of participants in this cluster (*n* = 18), but a majority of CP7 participants resided within a single neighborhood in the City of Salinas. A global Moran’s I test statistic for spatial autocorrelation of high cumulative pesticide-use near maternal residences resulted in a *p*-value < 0.001, suggesting that high amounts of cumulative pesticide-use was spatially clustered. Conversely, we did not observe spatial autocorrelation (clustering) for FSIQ scores for the children in our study, indicating that it is only the potential exposure levels in our study that exhibited statistically significant spatial clustering and not our outcome.

### 3.6. Variable Selection from BKMR

A full summary of the PIPs from the BKMR hierarchical variable selection are presented in the Supplementary Materials (Table S3). According to group PIP values, which represent the posterior probability of a pesticide class being included in the models, the OPs ranked highest, followed by pyrethroids > manganese-based fungicide > neonicotinoid > carbamates. The ranking of conditional PIPs within the OPs, which represents the probability of an individual pesticide being included into the models conditional on the OP group being selected, resulted in oxydemeton-methyl with the highest rank, followed by acephate > chlorpyrifos > dimethoate > naled > malathion > diazinon. For the pyrethroids conditional PIPs, permethrin ranked highest followed by cypermethrin > esfenvalerate > cyhalomethrin. Methomyl ranked higher compared to thiodicarb in terms of conditional PIPs for the carbamates, although carbamates overall had the lowest group PIP ranking (meaning carbamates were rarely selected into the model). Conditional PIPs for Mn-based fungicides and neonicotinoid pesticides are not presented since these classes included only a single pesticide each. A sensitivity analysis with BKMR hierarchical variable selection was performed, which heuristically varied the grouping of pesticides based on either the between-pesticide correlation structure or alternatively grouped by using a hierarchical clustering of variables method that systematically groups pesticides by their between-pesticide correlation structure, which resulted in a similar relative pesticide ranking of conditional PIPs as seen with the pesticide class-based grouping (Table S2). 

## 4. Discussion

Our primary aim was to use Bayesian profile regression (BPR) to characterize joint distributional patterns of correlated agricultural pesticide usage near pregnant women’s homes among a cohort residing in the Salinas Valley, CA and evaluate clustered pesticide profiles in relation to full scale IQ (FSIQ) in their children at seven years of age. The BPR partitioned the joint pesticide distributions effectively given that the analysis resulted in distinct pesticide profiles whether or not the outcome was included in the analysis. Including the outcome in the analysis, however, enabled us to characterize the association of different clusters of pesticide profiles with FSIQ while also accounting for model uncertainty in the Bayesian framework. We found that pesticide profiles with elevated joint distributions of multiple pesticides showed the strongest associations with deficits in childhood FSIQ (e.g., >1/2 SD from baseline group); conversely, pesticide profiles with joint pesticide-use distributions that were substantially lower (CP3 and CP6) were characterized by FSIQs that were above the study population’s overall mean FSIQ. Importantly, mapping of the pesticide use profiles suggested a distinct spatial dependency for the most hazardous pesticide profiles, along with evidence of positive spatial autocorrelation (clustering) in higher pesticide levels near maternal residences. 

While previous studies suggest that prenatal proximity to agricultural pesticide use for individual pesticides is associated with neurodevelopmental outcomes [12,13,41,61,62,63], ours is the first to explicitly show that it may be the combined exposure to multiple different pesticides and pesticide classes near maternal residences that may be of importance rather than nearby use of a single pesticide or single class of pesticides. Our findings also imply that studies investigating the neurodevelopmental effects of current-use agricultural pesticide exposures should analyze the pesticide mixtures as a whole, rather than using the more conventional single pollutant models typically applied [12,13,41,62,63], as such methods are unable to control for the influence of other covarying pesticides. 

Conventional multivariate regression struggles to reliably estimate combined effects for a large number of exposures within the context of highly correlated exposures. This observation is particularly true when the number of parameters are large and the number of observations are small, as is common in most cohort studies. For instance, in previous work by our group on this same cohort and using this same PUR data set using separate pesticide-specific regression models, we found adverse associations between single pesticides and FSIQ [12]. With conventional regression, we were previously unable to analyze multiple pesticide exposures jointly and thus unable to evaluate the potential for combined effects. In addition, while several pesticides in our previous work showed linear exposure-response relationships with FSIQ, the highly correlated nature of the pesticides meant that we could not disentangle the true magnitude of associations for each pesticide, which likely over-attributed the effect of a single pesticide. These challenges strongly support the need for a more advanced and diverse set of statistical approaches to investigate health effects from multiple pesticide exposures [20]. Implementing Bayesian clustering as a framework of analysis allowed us to evaluate potential joint patterns of multiple pesticide exposures, the spatial patterning of potential joint exposures, as well as adverse childhood cognitive associations with joint exposure to multiple correlated pesticides. 

Our findings lend support to the idea that exposure to mixtures of pesticides may have unanticipated effects on neurodevelopment in children. Given the results from our previous study mentioned above [12], and working under the assumption of additive effects of pesticides on FSIQ, we would expect to see substantially larger deficits in FSIQ between pesticide profiles with large differences in cumulative exposure levels, but this was not evidenced in our analysis. Pesticide profile cluster 1 (CP1) resulted in a cumulative pesticide-use profile that was more than two and half times higher than CP7 (670 kg vs. 235 kg), yet relative to the pesticide profile with the lowest cumulative pesticide-use (CP3) both CP1 and CP7 exhibited differences in ΔFSIQ (∆FSIQ_CP1_ = −6.9 vs. ∆FSIQ_CP7_ = −6.4, Table 3) that did not correspond to the magnitude of the difference in cumulative pesticide use levels. Additionally, after weighting the two clusters by their respective OP toxicity based on relative potency factor, as done in our previous study [12], we observe that CP1 is nearly two and half times higher (data not shown) in its estimated toxicity weighted use, yet this difference in apparent toxicity was also not reflected in our results. In addition, CP2 resulted in a cumulative (summed) pesticide use estimate that was slightly higher than that of CP7, yet the difference in FSIQ from the lowest pesticide profile cluster for CP2 was substantially different compared to that of CP7. A potentially important difference between these two clusters (CP7 and CP2) was that acephate and thiodicarb were classified as “very high” in CP7, whereas these two pesticides were only “moderately high” in CP2. This finding reveals the possibility that combined exposures to multiple pesticides may not result in assumed additive effects for each compound in a mixture [20], as has been seen in some toxicity studies [64,65]. This finding, however, should be taken with caution, since our study lacked a direct measure of prenatal exposure to pesticides. Also, unlike conventional regression models, profile regression is limited by the fact that it does not assume additivity of effects from multiple exposures because it partitions continuous joint exposure distribution into discrete clusters, which essentially represent latent categorical variables. This points towards an important limitation in using clustering-based methods, whereby some clusters may result in joint exposure distributions too wide to elucidate the effect of an individual chemical within a cluster on the outcome, especially where the signal is not particularly strong [22]. 

Given some of the limitations with profile regression already discussed, supplementary to profile regression, we also implemented Bayesian kernel machine regression (BKMR). Even though BKMR has its own set of limitations, the results indicate that oxydemeton-methyl, acephate, and maneb were particularly important pesticides in the observed exposure profile associations with FSIQ, and these same pesticides were elevated in the pesticide profile clusters that exhibited the largest deficits in FSIQ. While there is no clear guidance with BKMR in how to group exposures with respect to hierarchical variable selection, our sensitivity analysis of exposure groupings showed consistency in terms of the importance of these three pesticides with respect to showing the strongest associations with FSIQ.

Despite the numerous potentially neurotoxic pesticides evaluated in the present study, our analysis revealed only eight distinct pesticide-use pesticide profiles when including the outcome and nine pesticide profiles when excluding the outcome in the BPR analysis. This suggests that pesticide exposures in agricultural communities may occur in a relatively small number of discrete patterns of exposure, which could reflect the small number of different types of crops near maternal residences that have only a certain combination of pesticides applied. This finding, however, is consistent with a recent French study, wherein a set of pesticide mixtures that the French population is potentially exposed to was similarly identified using a Dirichlet process mixture model [66]. Using a combination of dietary and pesticide residue information on 79 different pesticides, researchers observed that just 25 pesticides contributed to the clustering, with a total of only seven exposure profiles observed from their analysis [66]. In a follow-up study, Crépet et al. [65] evaluated the toxicological effects of these seven different pesticide mixtures in vitro and found certain exposure mixtures effects went either beyond or below predicted additivity effects and that toxic effects were not readily predicted based on exposure to each individual compound within a pesticide profile. Our finding and those of Crépet et al. [65] highlight the potential value in examining joint pesticide exposure patterns to help prioritize specific “real-world” exposure-response combinations that can be tested in toxicological studies [65,67]. BPR could also be extended to take “real world” scenarios of exposures profiles to multiple pesticides to predict potentially adverse neurological effects [60].

A promising aspect of our analytic approach that is worthy of further exploration is determining the particular biologic drivers in the clustering of the pesticide profiles. Even though the correlation structure of pesticide use patterns clearly played an important role in how these profiles clustered together, it is less clear the degree to which the disease sub-model in BPR determined the clustering or whether biologic factors related to neurotoxicity may be important as well. Our sensitivity analysis suggested an important role for the outcome in partially determining the clustering patterns observed in our data (Table 4). Considering that the clustering of pesticide profiles was sensitive to the outcome and that pesticide-specific neurotoxic effects are inherently dependent on biologic pathways, the clustering observed in our study may be driven by similar or dissimilar mechanisms of toxicity, metabolism, or distribution for certain combinations of pesticides. For instance, the ordering of OP and carbamate anti-acetylcholinesterase activity, or possibly some other biologically plausible pathway, may counteract the cognitive effects of multiple neurotoxic pesticides to explain the potential for sub-additive effects [68]. Unfortunately, these questions of biologic drivers cannot be readily elucidated in our study data because we lacked the appropriate biomarkers of exposure. This area represents an important avenue for future research.

An important implication of our study points toward the value in examining spatial patterns of exposure profiles related to agricultural applications. Our mapping of pesticide profiles demonstrated that the profiles associated with the largest deficits in childhood FSIQ exhibited a distinct spatial pattern suggestive of spatial clustering in the southern Salinas Valley and along the outer border of the City of Salinas. Importantly, a test for spatial autocorrelation using Moran’s I test failed to reveal evidence within our study population of spatial clustering for lower FSIQ scores, signifying that spatial residual confounding in our outcome due to unmeasured sub-population characteristics is unlikely to explain the spatial patterns observed for the highest risk clusters. We further observed that the highest risk pesticide profiles tended to be on the outskirts of the towns closer to agricultural fields, especially for the City of Salinas. Hence, cluster analysis of pesticide-use patterns combined with spatial information on participants’ residences can be leveraged to map communities that are most likely to be disproportionately impacted by hazardous environmental chemical mixtures [21]. Such spatial information is potentially useful for stakeholders, including public policy makers, growers who apply pesticides, and members of the public potentially impacted by application of multiple pesticides to agricultural fields. This approach can also be extended for other purposes such as in evaluating the spatial patterns of hazardous air pollution mixtures [21]. 

We did not evaluate potentially neurotoxic agricultural herbicides or fumigants in our analysis of pesticide mixtures, which is an important limitation. Future studies, with larger sample sizes, are thus needed to evaluate a broader class of neurotoxic pesticides and other potentially neurotoxic chemicals. Another important limitation is a lack of validation of our pesticide exposure measure (i.e., PUR data) either with biomarkers or environmental measures for all of the pesticides considered. The application of pesticides near maternal residences during pregnancy does not necessarily mean the women were exposed to these pesticides during their pregnancy, or that the relative proportion of exposure is represented by the relative use of the active ingredients. In addition, the potential exposure to the pesticides considered in our study vary by application method and their physicochemical properties including volatility, degradation rates, deposition rates, and other characteristics that will ultimately determine their spatiotemporal fate and transport in the environment; yet these factors were not considered in our exposure assessment [69,70]. Thus, relying solely on residential proximity to reported pesticide applications can lead to exposure misclassification and potentially bias our findings towards the null. Several studies (including in the CHAMACOS cohort) demonstrate some positive correlations between nearby reported agricultural pesticide use based on PUR data and environmental contamination (i.e., house dust and outdoor air) or proximity to agricultural fields and pesticide metabolite levels in biological samples [30,31,32,33,35,71,72]. We were unable to fully account for other potential sources of pesticide exposure such as residential use of pesticides and ingestion of pesticide residues from fruits and vegetables. Although we controlled for total prenatal DAP concentrations, which is a strength of our study since it likely represents other sources (i.e., diet and take-home from resident farmworkers) of prenatal OP pesticide exposures that cannot be ascertained solely from PUR data, these exposure biomarkers have their own limitations. DAPs are non-specific to a particular OP, they do not include exposure to certain OPs such as acephate [73], they do not represent exposure to other non-OP pesticides, they do not represent long-term exposures [74], and they may reflect exposure to preformed metabolites and not simply their parent compounds [75]. Measured total prenatal DAPs were not correlated with any of the PUR pesticide use estimates, thus reinforcing the concept that total DAPs are likely to be representative of other OP pesticide exposure sources and that DAPs do not sufficiently represent long-term exposure levels. That being said, we removed DAPs from the model as a sensitivity analysis and did not see any substantive difference in our modelling results (data not shown). Furthermore, use of PUR data as a proxy of exposure allows us to evaluate health associations for pesticides that currently do not have a reliable biomarker of exposure. PUR data also allows us to evaluate which pesticides are driving the clustering and observed associations with health, as opposed to the non-specificity of DAPs, which obscures the variation in toxicity between OPs. There is a clear need to develop improved biomarkers with better specificity and sensitivity for a wider array of pesticides that represent long-term exposures.

Future work should attempt to overcome the limitations noted above, for instance, by improving characterization of pesticide exposure by developing predictive models based on measured pesticide concentrations in house dust, outdoor air, and personal samplers. Future work should also estimate proximity to neurotoxic pesticide use during the postnatal period using residential history information in addition to location of daycare facilities and schools that children attended. Furthermore, other chemical mixtures analytic frameworks may be applied to this or similar data sets to better characterize the possible contribution of individual pesticides to adverse neurologic effects while considering all exposures jointly [19,20]. Finally, we encourage other researchers to attempt to replicate our findings in other studies, especially in studies containing larger sample sizes.

## 5. Conclusions

We observed that pesticide-use profiles of neurotoxic pesticides used near the homes of pregnant women living in agricultural communities were associated with FSIQ deficits in their children. While this study lends supportive evidence to previous findings by our group and others, namely that proximity to agricultural applications of pesticides are associated with poorer neurodevelopmental outcomes in young children, the present study goes a step further by considering joint nearby pesticide use in relation to FSIQ to show potential sub-additive associations and demonstrates the spatial patterning of joint pesticide profiles of potential exposures. Epidemiologic analysis of pesticides and their potential health effects can benefit by taking a joint exposures approach to analysis and also by incorporating spatial patterning of joint distributions of potential exposures.

## Figures and Tables

**Table 1:** Summary statistics for estimates of agricultural pesticide use (kg) within one kilometer of maternal residence during pregnancy, Center for the Health Assessment of Mothers and Children of Salinas (CHAMACOS), 1999–2000 (*n* = 255).

Pesticide (Type)	Median (IQR)	Mean (SD)
Oxydemeton-methyl (OP)	10.59 (1.95, 24.18)	20.50 (34.89)
Acephate (OP)	9.16 (2.54, 28.90)	23.59 (41.99)
Chlorpyrifos (OP)	9.83 (1.23, 29.89)	24.10 (41.16)
Diazinon (OP)	22.22 (11.86, 50.59)	46.02 (70.47)
Malathion (OP)	1.57 (0, 12.71)	20.41 (49.58)
Dimethoate (OP)	3.47 (0.84, 16.12)	11.67 (17.04)
Naled (OP)	0.07 (0, 6.24)	6.24 (14.62)
Maneb (Mn)	55.20 (24.77, 123.60)	108.90 (149.56)
Methomyl (Carb)	10.00 (2.50, 28.07)	25.09 (40.13)
Thiodicarb (Carb)	0.45 (0, 1.67)	1.67 (3.14)
Permethrin (PR)	2.64 (1.03, 9.73)	7.34 (10.47)
Cypermethrin (PR)	0.007 (0, 0.47)	0.48 (1.05)
Cyhalothrin (PR)	0.13 (0.05, 0.42)	0.40 (0.80)
Esfenvalerate (PR)	0.15 (0.0003, 1.44)	1.43 (5.17)
Imidacloprid (N)	2.53 (1.37, 3.06)	5.14 (6.29)
Cumulative (Summed)	164.20 (67.53, 356.20)	303.00 (395.75)

OP = Organophosphate, Mn = Manganese-based fungicides, Carb = Carbamates, PR = Pyrethroid, N = Neonicotinoid, IQR = Interquartile Range, SD = Standard Deviation.

**Table 2:** Summary of empirical FSIQ scores (unadjusted) at age 7-year overall and by exposure profile clusters (*n* = 255).

Overall	*n*	Mean (95% CI) ^a^
	255	103.8 (102.1, 105.6)
**Cluster**		
CP1	59	98.6 (95.1, 102.2)
CP2	52	105.0 (101.2, 108.9)
CP3	50	106.7 (102.2, 111.1)
CP4	17	104.4 (97.0, 111.8)
CP5	43	104.8 (101.0, 108.7)
CP6	3	108.3 (84.7, 131.9)
CP7	18	101.4 (94.4, 108.4)
CP8	13	110.0 (102.6, 117.4)

CI = Confidence Interval. **^a^** Unadjusted means and confidence intervals for the entire study population and for each “hard” cluster derived from BPR.

**Table 3:** Summary of posterior distribution of FSIQ for each exposure profile cluster and the estimated difference in FSIQ compared to lowest exposure profile cluster at age 7-year resulting from the BPR (*n* = 255).

Clusters	Adjusted Posterior Mean FSIQ ^a,b^ (95% Credible Intervals)	Mean FSIQ_c from FSIQ_CP3 ^a,c^ (95% Credible Intervals)	Probability FSIQ_c < FSIQ_CP3 ^d^
CP1	95.5 (88.6, 102.0)	−6.9 (−11.7, −2.1)	0.998
CP2	100.1 (93.4, 106.3)	−2.4 (−7.2, 2.5)	0.83
CP3	102.5 (95.8, 108.7)	Ref	Ref
CP4	100.1 (91.8, 107.9)	−2.4 (−9.0, 4.3)	0.76
CP5	100.6 (93.1, 107.6)	−1.9 (−7.1, 3.3)	0.77
CP6	101.3 (93.2, 108.4)	−1.1 (−7.9, 3.4)	0.47
CP7	96.1 (87.7, 104.2)	−6.4 (−13.1, 0.5)	0.97
CP8	102.8 (94.1, 111.3)	0.3 (−7.0, 7.8)	0.47

**^a^** Adjusted for child’s age at WISC assessment (mean centered), sex, language of assessment, maternal education, maternal intelligence (mean centered), maternal country of birth, maternal depression at 7-year visit, HOME score at 7-year visit (mean centered), household poverty level at 7-year visit, and prenatal urinary dialkyl phosphate (DAPs) metabolites (log10, mean centered); **^b^** The posterior distribution of the expected FSIQ scores for the c^th^ cluster when holding fixed effect control variables at zero; **^c^** Difference between posterior distribution of expected FSIQ for the c^th^ cluster compared to posterior distribution of expected FSIQ for cluster 3 (reference group) when holding control variables at zero; **^d^** Probability that the posterior distribution of expected FSIQ for the c^th^ cluster is below the expected FSIQ for cluster 3 when holding control variables at zero.

**Table 4:** Comparison of best cluster assignment patterns when including (outcome feedback) or excluding (no outcome feedback) the outcome from the BPR analysis.

Clusters Excluding FSIQ Outcome in BPR										
Clusters Including FSIQ Outcome in BPR	CP1	CP2	CP3	CP4	CP5	CP6	CP7	CP8	CP9	Row Total
CP1	55	0	4	0	0	0	0	0	0	59
CP2	0	8	22	0	0	0	4	0	18	52
CP3	0	0	0	50	0	0	0	0	0	50
CP4	0	1	0	0	15	0	0	1	0	17
CP5	0	2	0	0	0	40	0	1	0	43
CP6	0	0	0	0	3	0	0	0	0	3
CP7	0	0	0	0	0	0	16	2	0	18
CP8	0	0	3	0	0	0	0	10	0	13
Column Total	55	11	29	50	18	40	20	14	18	255

## Supplementary Materials

The following are available online at www.mdpi.com/1660-4601/14/5/506/s1, Table S1: CHAMACOS study cohort characteristics (*n* = 255). WISC-IV, Wechsler Intelligence Scale for Children, 4th edition; HOME, Home Observation for Measurement of the Environment, Table S2: Summary of empirical FSIQ scores (unadjusted) at age 7-year overall and by exposure profile clusters (*n* = 255) after excluding the outcome from the profile regression, Table S3: Group and conditional posterior inclusion probabilities (PIP)s from BKMR using different pesticide groupings for hierarchical variable selection, Figure S1: Full posterior distributions of expected FSIQ and pesticide use estimates for each cluster. Expected FSIQ represents the estimated FSIQ when fixing control variables at zero across each sweep of the Markov chain Monte Carlo (MCMC) iterations. Boxplots for pesticides represent the distribution of quartile assignment probabilities across each sweep of the MCMC iterations. Red boxplots indicate that the distribution of probabilities is higher than the expected value (*p* = 0.25) for each quartile, green boxplots indicate that the distribution of probabilities is as would be expected (*p* = 0.25), and blue boxplots indicate that the distribution of probabilities is below what would be expected (*p* = 0.25).

## References

[1] Engel S.M., Berkowitz G.S., Barr D.B., Teitelbaum S.L., Siskind J., Meisel S.J., Wetmur J.G., Wolff M.S. (2007). Prenatal Organophosphate metabolite and organochlorine levels and performance on the brazelton neonatal behavioral assessment scale in a multiethnic pregnancy cohort. Am. J. Epidemiol..

[2] Eskenazi B., Kogut K., Huen K., Harley K.G., Bouchard M., Bradman A., Boyd-Barr D., Johnson C., Holland N. (2014). Organophosphate pesticide exposure, PON1, and neurodevelopment in school-age children from the CHAMACOS study. Environ. Res..

[3] Gaspar F.W., Harley K.G., Kogut K., Chevrier J., Mora A.M., Sjödin A., Eskenazi B. (2015). Prenatal DDT and DDE exposure and child IQ in the CHAMACOS cohort. Environ. Int..

[4] Horton M.K., Kahn L.G., Perera F., Barr D.B., Rauh V. (2012). Does the home environment and the sex of the child modify the adverse effects of prenatal exposure to chlorpyrifos on child working memory?. Neurotoxicol. Teratol..

[5] Lovasi G.S., Quinn J.W., Rauh V.A., Perera F.P., Andrews H.F., Garfinkel R., Hoepner L., Whyatt R., Rundle A. (2011). Chlorpyrifos exposure and urban residential environment characteristics as determinants of early childhood neurodevelopment. Am. J. Public Health.

[6] Rauh V.A., Perera F.P., Horton M.K., Whyatt R.M., Bansal R., Hao X., Liu J., Barr D.B., Slotkin T.A., Peterson B.S. (2012). Brain anomalies in children exposed prenatally to a common organophosphate pesticide. Proc. Natl. Acad. Sci. USA.

[7] Rauh V., Arunajadai S., Horton M., Perera F., Hoepner L., Barr D.B., Whyatt R. (2011). Seven-year neurodevelopmental scores and prenatal exposure to chlorpyrifos, a common agricultural pesticide. Environ. Health Perspect..

[8] Ribas-Fito N., Torrent M., Carrizo D., Munoz-Ortiz L., Julvez J., Grimalt J.O., Sunyer J. (2006). In utero exposure to background concentrations of DDT and cognitive functioning among preschoolers. Am. J. Epidemiol..

[9] Viel J.-F., Warembourg C., Le Maner-Idrissi G., Lacroix A., Limon G., Rouget F., Monfort C., Durand G., Cordier S., Chevrier C. (2015). Pyrethroid insecticide exposure and cognitive developmental disabilities in children: The PELAGIE mother–child cohort. Environ. Int..

[10] Young J.G., Eskenazi B., Gladstone E.A., Bradman A., Pedersen L., Johnson C., Barr D.B., Furlong C.E., Holland N.T. (2005). Association between in utero organophosphate pesticide exposure and abnormal reflexes in neonates. NeuroToxicology.

[11] Bouchard M.F., Chevrier J., Harley K.G., Kogut K., Vedar M., Calderon N., Trujillo C., Johnson C., Bradman A., Barr D.B. (2011). Prenatal exposure to organophosphate pesticides and IQ in 7-year-old children. Environ. Health Perspect..

[12] Gunier R.B., Bradman A., Harley K.G., Kogut K., Eskenazi B. (2016). Prenatal residential proximity to agricultural pesticide use and IQ in 7-year-old children. Environ. Health Perspect..

[13] Roberts E.M., English P.B., Grether J.K., Windham G.C., Somberg L., Wolff C. (2007). Maternal residence near agricultural pesticide applications and autism spectrum disorders among children in the California Central Valley. Environ. Health Perspect..

[14] Zaunbrecher V., Hattis D., Melnick R., Kegley S., Malloy T., Froines J. (2016). Exposure and Interaction: The Potential Health Impacts of Using Multiple Pesticides.

[15] Moser V.C., Simmons J.E., Gennings C. (2006). Neurotoxicological interactions of a five-pesticide mixture in preweanling rats. Toxicol. Sci. Off. J. Soc. Toxicol..

[16] Rizzati V., Briand O., Guillou H., Gamet-Payrastre L. (2016). Effects of pesticide mixtures in human and animal models: An update of the recent literature. Chem. Biol. Interact..

[17] Hernández A.F., Parrón T., Tsatsakis A.M., Requena M., Alarcón R., López-Guarnido O. (2013). Toxic effects of pesticide mixtures at a molecular level: Their relevance to human health. Toxicology.

[18] Moser V.C., Padilla S., Simmons J.E., Haber L.T., Hertzberg R.C. (2012). Impact of chemical proportions on the acute neurotoxicity of a mixture of seven carbamates in preweanling and adult rats. Toxicol. Sci..

[19] Bobb J.F., Valeri L., Claus Henn B., Christiani D.C., Wright R.O., Mazumdar M., Godleski J.J., Coull B.A. (2015). Bayesian kernel machine regression for estimating the health effects of multi-pollutant mixtures. Biostatistics.

[20] Braun J.M., Gennings C., Hauser R., Webster T.F. (2016). What Can Epidemiological studies tell us about the impact of chemical mixtures on human health?. Environ. Health Perspect..

[21] Coker E., Liverani S., Ghosh J.K., Jerrett M., Beckerman B., Li A., Ritz B., Molitor J. (2016). Multi-pollutant exposure profiles associated with term low birth weight in Los Angeles County. Environ. Int..

[22] Coull B.A., Bobb J.F., Wellenius G.A., Kioumourtzoglou M.-A., Mittleman M.A., Koutrakis P., Godleski J.J. (2015). Part 1. Statistical learning methods for the effects of multiple air pollution constituents. Res. Rep. Health Eff. Inst..

[23] Molitor J., Papathomas M., Jerrett M., Richardson S. (2010). Bayesian profile regression with an application to the national survey of children’s health. Biostatistics.

[24] Oakes M., Baxter L., Long T.C. (2014). Evaluating the application of multipollutant exposure metrics in air pollution health studies. Environ. Int..

[25] Park E.S., Symanski E., Han D., Spiegelman C. (2015). Part 2. Development of enhanced statistical methods for assessing health effects associated with an unknown number of major sources of multiple air pollutants. Res. Rep. Health Eff. Inst..

[26] Pirani M., Best N., Blangiardo M., Liverani S., Atkinson R.W., Fuller G.W. (2015). Analysing the health effects of simultaneous exposure to physical and chemical properties of airborne particles. Environ. Int..

[27] Zanobetti A., Austin E., Coull B.A., Schwartz J., Koutrakis P. (2014). Health effects of multi-pollutant profiles. Environ. Int..

[28] CDPR (2016). Summary of Pesticide Use Report Data 2014 Indexed by Chemical.

[29] CDPR Pesticide Use Reporting. http://www.cdpr.ca.gov/docs/pur/purmain.htm.

[30] Gunier R.B., Ward M.H., Airola M., Bell E.M., Colt J., Nishioka M., Buffler P.A., Reynolds P., Rull R.P., Hertz A. (2011). Determinants of agricultural pesticide concentrations in carpet dust. Environ. Health Perspect..

[31] Harnly M., McLaughlin R., Bradman A., Anderson M., Gunier R. (2005). Correlating agricultural use of organophosphates with outdoor air concentrations: A particular concern for children. Environ. Health Perspect..

[32] Harnly M.E., Bradman A., Nishioka M., McKone T.E., Smith D., McLaughlin R., Kavanagh-Baird G., Castorina R., Eskenazi B. (2009). Pesticides in dust from homes in an agricultural area. Environ. Sci. Technol..

[33] Li L., Johnson B., Segawa R. (2005). Empirical relationship between use, area, and ambient air concentration of methyl bromide. J. Environ. Qual..

[34] Bradman A., Eskenazi B., Barr D.B., Bravo R., Castorina R., Chevrier J., Kogut K., Harnly M.E., McKone T.E. (2005). Organophosphate urinary metabolite levels during pregnancy and after delivery in women living in an agricultural community. Environ. Health Perspect..

[35] Bradman A., Castorina R., Boyd Barr D., Chevrier J., Harnly M.E., Eisen E.A., McKone T.E., Harley K., Holland N., Eskenazi B. (2011). Determinants of organophosphorus pesticide urinary metabolite levels in young children living in an agricultural community. Int. J. Environ. Res. Public Health.

[36] Bigelow J.L., Dunson D.B. (2009). Bayesian semiparametric joint models for functional predictors. J. Am. Stat. Assoc..

[37] Chung Y., Dunson D.B. (2009). Nonparametric bayes conditional distribution modeling with variable selection. J. Am. Stat. Assoc..

[38] Dunson D.B. (2009). Nonparametric Bayes local partition models for random effects. Biometrika.

[39] Dunson D.B., Herring A.H., Siega-Riz A.M. (2008). Bayesian inference on changes in response densities over predictor clusters. J. Am. Stat. Assoc..

[40] Eskenazi B., Bradman A., Castorina R. (1999). Exposures of children to organophosphate pesticides and their potential adverse health effects. Environ. Health Perspect..

[41] Rowe C., Gunier R., Bradman A., Harley K.G., Kogut K., Parra K., Eskenazi B. (2016). Residential proximity to organophosphate and carbamate pesticide use during pregnancy, poverty during childhood, and cognitive functioning in 10-year-old children. Environ. Res..

[42] Lonare M., Kumar M., Raut S., Badgujar P., Doltade S., Telang A. (2014). Evaluation of imidacloprid-induced neurotoxicity in male rats: A protective effect of curcumin. Neurochem. Int..

[43] Gill K.D., Flora G., Pachauri V., Flora S.J.S., Satoh T., Gupta R.C. (2011). Neurotoxicity of organophosphates and carbamates. Anticholinesterase Pesticides.

[44] Costello S., Cockburn M., Bronstein J., Zhang X., Ritz B. (2009). Parkinson’s disease and residential exposure to maneb and paraquat from agricultural applications in the central valley of California. Am. J. Epidemiol..

[45] DeMicco A., Cooper K.R., Richardson J.R., White L.A. (2010). Developmental neurotoxicity of pyrethroid insecticides in zebrafish embryos. Toxicol. Sci..

[46] Johnstone A.F.M., Strickland J.D., Crofton K.M., Gennings C., Shafer T.J. (2016). Effects of an environmentally-relevant mixture of pyrethroid insecticides on spontaneous activity in primary cortical networks on microelectrode arrays. NeuroToxicology.

[47] Wechsler D. (2003). Wechsler Intelligence Scale for Children.

[48] Woodcock R., Johnson M. (1990). Woodcock-Johnson Psycho-Educational Battery-Revised.

[49] Sugland B.W., Zaslow M., Smith J.R., Brooks-Gunn J., Coates D., Blumenthal C., Moore K.A., Griffin T., Bradley R. (1995). The Early Childhood HOME inventory and HOME-short form in differing racial/ethnic groups: Are there differences in underlying structure, internal consistency of subscales, and patterns of prediction?. J. Fam. Issues.

[50] Dunn M., Dunn L.M. (1981). Peabody Picture Vocabulary Test—Revised.

[51] Gilks W.R., Richardson S., Spiegelhalter D.J., Gilks W.R., Richardson S., Spiegelhalter D.J. (1998). Markov Chain Monte Carlo in Practice.

[52] Jain S., Neal R.M. (2007). Splitting and merging components of a nonconjugate Dirichlet process mixture model. Bayesian Anal..

[53] Liverani S., Hastie D.I., Azizi L., Papathomas M., Richardson S. (2015). PReMiuM: An R package for profile regression mixture models using dirichlet processes. J. Stat. Softw..

[54] Hastie D.I., Liverani S., Azizi L., Richardson S., Stücker I. (2013). A semi-parametric approach to estimate risk functions associated with multi-dimensional exposure profiles: Application to smoking and lung cancer. BMC Med. Res. Methodol..

[55] Papathomas M., Molitor J., Richardson S., Riboli E., Vineis P. (2010). Examining the joint effect of multiple risk factors using exposure risk profiles: Lung cancer in nonsmokers. Environ. Health Perspect..

[56] National Research Council (1997). Environmental Epidemiology.

[57] Papathomas M., Molitor J., Hoggart C., Hastie D., Richardson S. (2012). Exploring data from genetic association studies using bayesian variable selection and the dirichlet process: Application to searching for gene × Gene patterns: Searching for gene × gene patterns. Genet. Epidemiol..

[58] Greenland S. (1993). Methods for epidemiologic analyses of multiple exposures: A review and comparative study of maximum-likelihood, preliminary-testing, and empirical-bayes regression. Stat. Med..

[59] Thomas D.C., Witte J.S., Greenland S. (2007). Dissecting effects of complex mixtures: Who? Afraid of informative priors?. Epidemiology.

[60] Liverani S., Lavigne A., Blangiardo M. (2016). Modelling collinear and spatially correlated data. Spat. Spatio-Temporal Epidemiol..

[61] Rull R.P., Ritz B., Shaw G.M. (2006). Neural tube defects and maternal residential proximity to agricultural pesticide applications. Am. J. Epidemiol..

[62] Shelton J.F., Geraghty E.M., Tancredi D.J., Delwiche L.D., Schmidt R.J., Ritz B., Hansen R.L., Hertz-Picciotto I. (2014). Neurodevelopmental disorders and prenatal residential proximity to agricultural pesticides: The CHARGE study. Environ. Health Perspect..

[63] Yang W., Carmichael S.L., Roberts E.M., Kegley S.E., Padula A.M., English P.B., Shaw G.M. (2014). Residential agricultural pesticide exposures and risk of neural tube defects and orofacial clefts among offspring in the San Joaquin Valley of California. Am. J. Epidemiol..

[64] Cedergreen N. (2014). Quantifying synergy: A systematic review of mixture toxicity studies within environmental toxicology. PLoS ONE.

[65] Crépet A., Héraud F., Béchaux C., Gouze M.E., Pierlot S., Fastier A., Leblanc J.C., Le Hégarat L., Takakura N., Fessard V. (2013). The PERICLES research program: An integrated approach to characterize the combined effects of mixtures of pesticide residues to which the French population is exposed. Toxicology.

[66] Crépet A., Tressou J., Graillot V., Béchaux C., Pierlot S., Héraud F., Leblanc J.C. (2013). Identification of the main pesticide residue mixtures to which the French population is exposed. Environ. Res..

[67] Carlin D.J., Rider C.V., Woychik R., Birnbaum L.S. (2013). Unraveling the health effects of environmental mixtures: An NIEHS priority. Environ. Health Perspect..

[68] Colovic M.B., Krstic D.Z., Lazarevic-Pasti T.D., Bondzic A.M., Vasic V.M. (2013). Acetylcholinesterase inhibitors: Pharmacology and toxicology. Curr. Neuropharmacol..

[69] Armon R.H., Hänninen O., Armon R.H., Hänninen O. (2015). Environmental Indicators.

[70] Vermeire T., McPhail R., Waters M. D. (2001). Organophosphorous Pesticides in the Environment.

[71] Davidson C. (2003). California Amphibian Declines and Historic Pesticide Use.

[72] Zhan Y., Zhang M. (2014). Spatial and temporal patterns of pesticide use on California almonds and associated risks to the surrounding environment. Sci. Total Environ..

[73] Curl C.L., Beresford S.A.A., Fenske R.A., Fitzpatrick A.L., Lu C., Nettleton J.A., Kaufman J.D. (2015). Estimating pesticide exposure from dietary intake and organic food choices: The Multi-Ethnic Study of Atherosclerosis (MESA). Environ. Health Perspect..

[74] Bradman A., Kogut K., Eisen E.A., Jewell N.P., Quirós-Alcalá L., Castorina R., Chevrier J., Holland N.T., Barr D.B., Kavanagh-Baird G. (2013). Variability of organophosphorous pesticide metabolite levels in spot and 24-h urine samples collected from young children during 1 week. Environ. Health Perspect..

[75] Zhang X., Driver J.H., Li Y., Ross J.H., Krieger R.I. (2008). Dialkylphosphates (DAPs) in fruits and vegetables may confound biomonitoring in organophosphorus insecticide exposure and risk assessment. J. Agric. Food Chem..

