# Supplementary material for: Association between Pesticide Profiles Used on Agricultural Fields near Maternal Residences during Pregnancy and IQ at Age 7 Years
Source: Int J Environ Res Public Health. 2017 May 9;14(5):506. doi: 10.3390/ijerph14050506 (PMC5451957; doi:10.3390/ijerph14050506)
Supplement: Supplementary file 1 [file ijerph-14-00506-s001.pdf]

Supplementary Materials: Association between Pesticide Profiles Used on Agricultural Fields near Maternal Residences during Pregnancy and IQ at Age 7 Years

Eric Coker, Robert Gunier, Asa Bradman, Kim Harley, Katherine Kogut, John Molitor and Brenda Eskenazi

Table S1. CHAMACOS study cohort characteristics (*n* = 255). WISC-IV, Wechsler Intelligence Scale for Children, 4th edition; HOME, Home Observation for Measurement of the Environment.

**Cohort Characteristic**	***n* (%) or Mean (SD)**
Maternal Country of Birth	
Mexico	222 (87.1)
United States and other	33 (12.9)
Maternal Education	
≤6th grade	121 (47.5)
7th grade or more	134 (52.6)
Family income at 7-year visit	
<Poverty level	180 (70.6)
≥Poverty level	75 (29.41)
Maternal depression at 7-year visit	
Yes	71 (27.8)
No	184 (72.2)
Sex	
Girl	136 (53.3)
Boy	119 (46.7)
Language of WISC-IV tests	
Spanish	171 (67.1)
English	84 (32.9)
Child age at WISC-IV assessment (years)	7.1 (0.25)
HOME score at 7-year visit	17.8 (2.8)
Maternal Peabody Picture Vocabulary Test score at 6 months postpartum	85.7 (20.8)

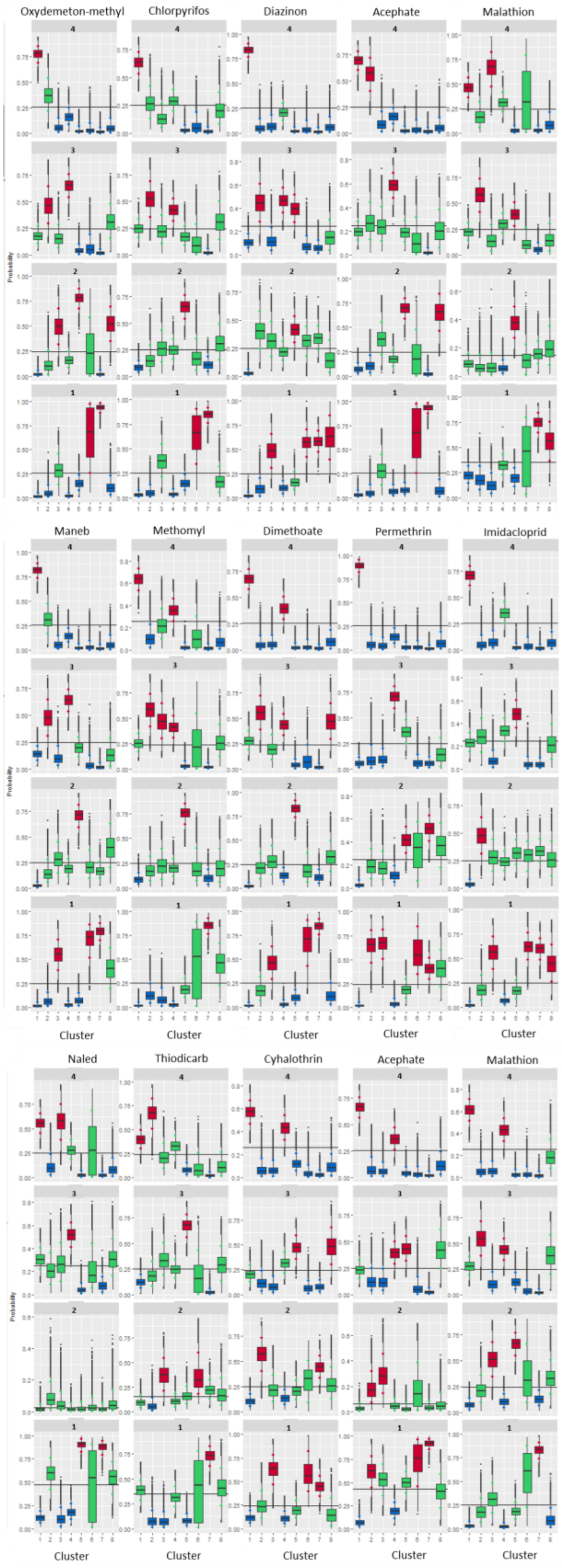

Figure S1. Full posterior distributions of expected FSIQ and pesticide use estimates for each cluster. Expected FSIQ represents the estimated FSIQ when fixing control variables at zero across each sweep of the Markov chain Monte Carlo (MCMC) iterations. Boxplots for pesticides represent the distribution of quartile assignment probabilities across each sweep of the MCMC iterations. Red boxplots indicate that the distribution of probabilities is higher than the expected value (p = 0.25) for each quartile, green boxplots indicate that the distribution of probabilities is as would be expected (p = 0.25), and blue boxplots indicate that the distribution of probabilities is below what would be expected (p = 0.25).

Table S2. Summary of empirical FSIQ scores (unadjusted) at age 7-years overall and by exposure profile clusters (n = 255) after excluding the outcome from the profile regression.

Cluster Profiles	Mean FSIQ	Confidence Intervals of FSIQ		Fixed Effects Only Model				Random Effects Model
		Lower 95th Percentile	Upper 95th Percentile	β [a]	Lower 95th Percentile	Upper 95th Percentile	p-Value	ΔFSIQ (SE) [a,b]
CP1	96.64	89.12	104.15	−6.82	−11.88	−1.77	0.008	−1.88 (1.19)
CP2	97.03	87.12	106.94	−6.43	−14.87	2.01	0.14	−0.56 (1.53)
CP3	103.16	95.40	110.91	−0.30	−6.22	5.61	0.92	0.83 (1.36)
CP4	103.46	96.36	110.56	Reference				1.24 (1.22)
CP5	102.13	93.26	111.01	−1.33	−8.33	5.67	0.71	0.36 (1.46)
CP6	101.67	93.64	109.70	−1.79	−7.26	3.67	0.52	0.43 (1.28)
CP7	98.77	89.85	107.68	−4.69	−11.51	2.12	0.18	−0.45 (1.44)
CP8	100.98	91.68	110.27	−2.48	−10.13	5.16	0.53	0.07 (1.50)
CP9	100.42	91.85	108.99	−3.04	−9.97	3.89	0.39	−0.04 (1.46)

[a] Adjusted for child's age at WISC assessment (mean centered), sex, language of assessment, maternal education, maternal intelligence (mean centered), maternal country of birth, maternal depression at 7-year visit, HOME score at 7-year visit (mean centered), household poverty level at 7-year visit. and prenatal urinary DAPs (log10, mean centered). [b] Pesticide profile clusters were fit as random effects using the *lmer* function in R to smooth effect estimates and avoid multiple testing comparisons. Likelihood ratio test p-value < 0.0001.

Table S3. Group and conditional posterior inclusion probabilities (PIP)s from BKMR using different pesticide groupings for hierarchical variable selection [a].

Pesticide	Pesticide Class	Group PIP [b]	Conditional PIP	Group PIP [c]	Conditional PIP	Group PIP [d]	Conditional PIP	Group PIP [c]	Conditional PIP
Thiodicarb	C	0.185	0.462	0.230	0.391	0.567	0.012	0.189	1.000
Methomyl	C	0.185	0.538	0.832	0.015	0.567	0.017	0.584	0.029
Maneb	M	0.391	1	0.832	0.162	0.567	0.147	0.584	0.147
Imidacloprid	N	0.235	1	0.832	0.021	0.567	0.039	0.584	0.041
Malathion	OP	0.436	0.05	0.230	0.311	0.140	0.501	0.197	0.595
Naled	OP	0.436	0.051	0.230	0.298	0.140	0.499	0.197	0.405
Acephate	OP	0.436	0.319	0.832	0.240	0.567	0.257	0.584	0.272
Oxydemeton-methyl	OP	0.436	0.369	0.832	0.229	0.567	0.288	0.584	0.286
Dimethoate	OP	0.436	0.074	0.832	0.028	0.567	0.050	0.584	0.045
Chlorpyrifos	OP	0.436	0.088	0.832	0.026	0.567	0.035	0.584	0.033
Diazinon	OP	0.436	0.05	0.832	0.014	0.567	0.033	0.584	0.032
Permethrin	P	0.413	0.327	0.832	0.109	0.567	0.122	0.584	0.115
Cypermethrin	P	0.413	0.29	0.832	0.084	0.444	0.457	0.422	0.212
Esfenvalerate	P	0.413	0.237	0.832	0.049	0.444	0.338	0.422	0.454
Cyhalothrin	P	0.413	0.146	0.832	0.024	0.444	0.205	0.422	0.334

[a] The different colors are indicating which pesticides were grouped together in the BKMR analysis. For instance, the blue color for the first Group PIP column indicates that Thiodicarb and Methomyl were grouped together in the BKMR analysis. [b] Groups defined by pesticide class. [c] Groups defined using heuristic evaluating between pesticide correlations. [d] Groups defined using hierarchical clustering of variables with consolidation methodology.

© 2017 by the authors; licensee MDPI, Basel, Switzerland. This article is an open access article distributed under the terms and conditions of the Creative Commons by Attribution (CC-BY) license (http://creativecommons.org/licenses/by/4.0/).
